# Focal Adhesion Kinase (FAK) Inhibition Synergizes with KRAS G12C Inhibitors in Treating Cancer through the Regulation of the FAK–YAP Signaling

**DOI:** 10.1002/advs.202100250

**Published:** 2021-06-20

**Authors:** Baoyuan Zhang, Yan Zhang, Jiangwei Zhang, Ping Liu, Bo Jiao, Zaiqi Wang, Ruibao Ren

**Affiliations:** ^1^ Shanghai Institute of Hematology State Key Laboratory for Medical Genomics National Research Center for Translational Medicine International Center for Aging and Cancer Collaborative Innovation Center of Hematology Ruijin Hospital Shanghai Jiao Tong University School of Medicine Shanghai 200025 China; ^2^ InxMed (Shanghai) Co., Ltd Shanghai 201202 China

**Keywords:** drug resistance, FAK, KRAS G12C, synergy, YAP

## Abstract

KRAS mutation is one of the most prevalent genetic drivers of cancer development, yet KRAS mutations are until very recently considered undruggable. There are ongoing trials of drugs that target the KRAS G12C mutation, yet acquired drug resistance from the extended use has already become a major concern. Here, it is demonstrated that KRAS G12C inhibition induces sustained activation of focal adhesive kinase (FAK) and show that a combination therapy comprising KRAS G12C inhibition and a FAK inhibitor (IN10018) achieves synergistic anticancer effects. It can simultaneously reduce the extent of drug resistance. Diverse CDX and PDX models of KRAS G12C mutant cancer are examined and synergistic benefits from the combination therapy are consistently observed. Mechanistically, it is found that both aberrant FAK–YAP signaling and FAK‐related fibrogenesis impact on the development of KRAS G12C inhibitor resistance. This study thus illustrates the mechanism of resistance of cancer to the treatment of KRAS G12C inhibitor, as well as an innovative combination therapy to improve treatment outcomes for KRAS G12C mutant cancers.

## Graphical abstract

Here, a therapeutic strategy is described for KRAS G12C mutant cancers by the combination of KRAS G12C and FAK inhibition. Mechanistically, the aberrant FAK‐YAP signaling hampers benefits from KRAS G12C inhibitors. The combination of KRAS G12C and FAK inhibitors produces synergistic antitumor effects, providing a regimen to obtain strengthened and prolonged treatment outcomes for KRAS G12C mutant cancers.

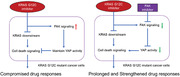

## 1 Introduction

RAS oncogenes are frequently mutated in human cancers, with mutations in three isoforms KRAS, NRAS, and HRAS having the prevalence, with KRAS alone accounting for the pathogenesis of up to 13% of cancers.^[^ ^1^ ^]^ For example, around 30% of nonsmall cell lung cancers (NSCLC), 40% of colorectal cancers (CRC), and 80% of pancreatic cancers harbor KRAS mutations, emphasizing its driver role in cancer development.^[^ ^2^ ^]^ KRAS was once considered to be undruggable since it was exceedingly challenging to identify actionable target‐inhibitor binding sites.^[^ ^3^ ^]^ However, a recent strategy targeting the very common KRAS 12 glycine to cysteine (G12C) mutation proved successful, based on covalent modification of the cysteine residue.^[^ ^4^ ^]^ This strategy was translated into a series of KRAS G12C inhibitors including AMG510 and MRTX849, both of which are currently in clinical trials.^[^ ^5^ ^]^ Although these KRAS G12C inhibitors are showing promising antitumor activity, the fact that they are targeted therapies inevitably means they are vulnerable to the development of intrinsic or adaptive resistance;^[^ ^6^, ^7^ ^]^ such resistance can severely limit the long‐term therapeutic utility of these treatments. Despite the relatively short history of KRAS G12C inhibitors, there are already multiple reports that abnormal KRAS vertical signaling contributes to the development of drug resistance against these agents,^[^ ^8^, ^9^ ^]^ Beyond confirming long‐understood impacts from the use of targeted therapies, these findings have suggested that targeting KRAS adjacent signaling components help to provide sustained beneficial clinical outcomes.

Focal adhesive kinase (FAK) is a nonreceptor kinase that exerts functions on regulation of cell growth, cell scaffold dynamics, and signal transduction.^[^ ^10^ ^]^ Elevated levels of the phosphorylated (active) form of FAK are associated with poor prognosis in multiple cancers, and multiple FAK inhibitors have been developed and tested as anticancer agents in clinical trials.^[^ ^11^ ^]^ FAK acts downstream of KRAS, and its inhibition is effective in suppressing the progression of KRAS mutant cancer.^[^ ^12^, ^13^, ^14^ ^]^ Besides, these direct links to KRAS, it is intriguing that activation of FAK signaling has been proposed as a mechanism underlying resistance to target therapies, specifically because of its impacts on multiple aspects of the tumor microenvironment.^[^ ^15^, ^16^ ^]^ This multifaceted tumor‐related functionality of FAK motivated our scientific and medical interest about whether combining KRAS G12C and FAK inhibition may produce synergistic effects. We were also interested in whether such synergism may help in overcoming the drug resistance that challenges the proper stewardship and deployment of the currently available KRAS G12C inhibitors.

In the current study, we found that FAK signaling activity is adaptively induced upon KRAS G12C inhibition. The combination of KRAS G12C inhibitors (AMG510 or MRTX849) alongside a clinical‐stage small molecule FAK inhibitor IN10018 produced encouraging anti‐cancer effects against multiple cancer cell lines, CDX, and PDX models of KRAS G12C mutant cancers. We also demonstrate that the FAK–YAP axis compromises the long‐term drug effects of KRAS G12C inhibitors. After showing that FAK inhibition or YAP knockdown obviously enhanced cancer cell killing outcomes when combined with KRAS G12C inhibitors treatment, our data from testing with diverse different CDX and PDX models showed that AMG510 treatment as a monotherapy resulted in FAK‐related excessive tumor fibrosis, a frequent cause underlying acquired drug resistance.^[^ ^17^, ^18^ ^]^ IN10018 efficiently eliminated the fibrogenesis, and conferred synergistic effects, substantially outperforming the tumor growth inhibition effects of AMG510 monotherapy. Thus, our study demonstrates how combination therapies comprising KRAS G12C and FAK inhibitors achieve synergistic anticancer effects while simultaneously reducing acquired drug resistance, potentially maximizing the treatment outcomes for cancers harboring KRAS G12C mutations.

## 2 Results

### 2.1 FAK Serves as an Informative Biomarker for Aberrant KRAS Signaling and Its Activation is Adaptively Induced Upon KRAS G12C Inhibition

**Figure 1:**
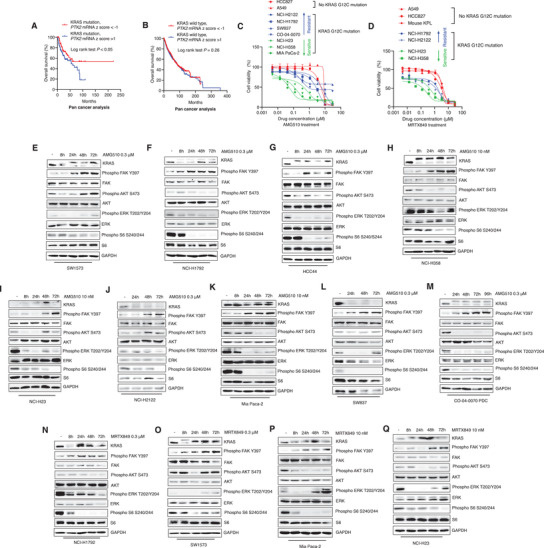
FAK serves as an informative biomarker for aberrant KRAS signaling and its activation is adaptively induced upon KRAS G12C inhibition. A) TCGA survival analysis for KRAS mutant cancer patients based on *PTK*2 expression levels. (KRAS mutation, *PTK*2 mRNA *z* score < ‐1: *n* = 87; KRAS mutation, *PTK*2 mRNA *z* score > 1: *n* = 107). Log rank test was performed for statistical analysis, *P* values are shown. B) TCGA survival analysis for KRAS wildtype cancer patients based on *PTK*2 expression levels. (KRAS wildtype, *PTK*2 mRNA *z* score < ‐1: *n* = 1331; KRAS wildtype, *PTK*2 mRNA *z* score > 1, *n* = 1398). Log‐rank test was performed for statistical analysis, *P* value is shown. C) Cell viability tests for AMG510 on different cancer cell lines. The cell lines were treated with different doses of AMG510 for 72 h. Finally, CTG assay was performed for cell viability evaluation. (Data represent mean ± SEM, *n* ≥ 3). D) Cell viability tests for MRTX849 on different cancer cell lines. The cell lines were treated with different doses of MRTX849 for 72 h. CTG assay was performed for cell viability evaluation. (Data represent mean ± SEM, *n* ≥ 3). E–M) Western blot analysis for FAK signaling and downstream markers of KRAS upon the treatment of AMG510 for different time points. N–Q) Western blot analysis for FAK signaling and downstream markers of KRAS upon the treatment of MRTX849 for different time points.

TCGA‐cancer survival data were divided into KRAS wild type and KRAS mutation subgroups.^[^ ^19^ ^]^ Within the KRAS mutant subgroup, lower RNA expression of *PTK*2 (FAK coding gene) correlates with better survival outcomes, while for KRAS wild type subgroup, there is no difference between the *PTK*2 low and high expression groups (**Figure** **1**A,B), suggesting that FAK may be an informative biomarker for aberrant KRAS signaling induced cancer development.

We then performed cell viability assay for AMG510, and MRTX849 in different cancer types (NSCLC, CRC, and pancreatic cancer) including KRAS wild type, KRAS non‐G12C mutation, and KRAS G12C mutation cell lines. Some of the KRAS G12C mutant cell lines showed good sensitivity to KRAS G12C inhibition (e.g., Mia PaCa‐2 and NCI‐H358), while other cell lines were not obviously impacted by either treatment, suggesting intrinsic resistance (Figure 1C,D).

The nontoxic doses for each cell line were then used in experiments based on immunoblotting to identify the involvement of KRAS downstream signaling components. Similar to previous reports,^[^ ^8^, ^20^ ^]^ KRAS G12C inhibition alone by either AMG510 or MRTX849 resulted in decreased activities for PI3K/AKT, MAPK, and mTOR signaling pathways to differing degrees for various KRAS G12C mutant cell lines (Figure 1E–Q). We did note that some of the cell lines exhibited rebounds in the accumulation of downstream KRAS signaling components upon time‐course treatment. Further, these experiments revealed a clear band shift for the KRAS protein in all the KRAS G12C mutant cell lines, indicating that KRAS G12C inhibitors exert effects after drug administration.^[^ ^21^ ^]^ We also noted that Phospho FAK Y397 exhibited sustained stimulation in all the tested cell lines, indicating its potential function in adaptive drug resistance to KRAS G12C inhibition (Figure 1E–Q). A549 and HCC827 cells which are KRAS non‐G12C mutant cell lines were also treated with AMG510 or MRTX849, no stimulation of KRAS downstream signaling components including FAK signaling was observed for either cell line (Figure S1A–C, Supporting Information).

### 2.2 The Small Molecule FAK Inhibitor IN10018 Confers Potent Anticancer Effects against Diverse KRAS Mutant Cell Lines as well as CDX and PDX Tumor Models

We in silico analyzed the essentiality of *PTK*2 for the growth of KRAS mutation‐dependent cell lines identified by Achilles’ project (Figure S2A, Supporting Information).^[^ ^22^ ^]^ Analysis of data for shRNA modulated *PTK*2 knockdown showed that, unlike any of the KRAS wild‐type cancer cell lines, all of the KRAS‐mutant‐dependent cell lines exhibited growth modulation related to *PTK*2 expression (Figure S2B, Supporting Information). The small molecule FAK inhibitor IN10018 was formerly developed by Boehringer‐Ingelheim (BI) under the name BI853520;^[^ ^23^ ^]^ it is a highly selective clinical‐stage FAK inhibitor.^[^ ^23^, ^24^ ^]^ Based on our analysis and other reports,^[^ ^12^, ^14^ ^]^ we hypothesized that FAK inhibition by IN10018 may exert a good anticancer response to KRAS mutant cancer cells and tumors.

**Figure 2:**
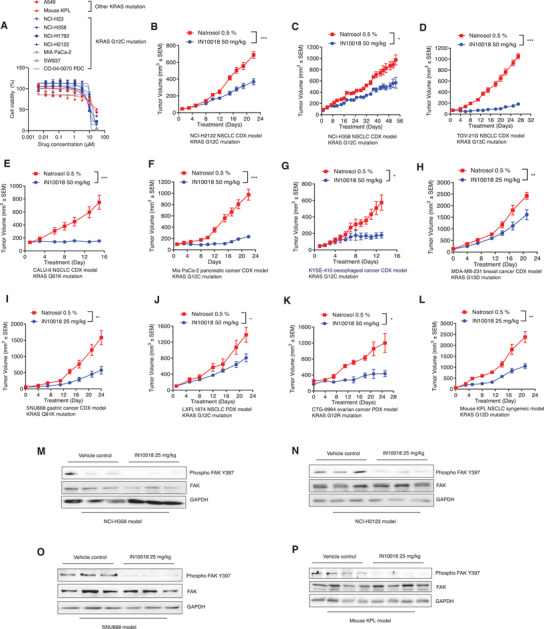
The small molecule FAK inhibitor IN10018 confers potent anti‐cancer effects against diverse KRAS mutant cell lines as well as CDX and PDX tumor models. A) Cell viability assay for the cell lines treated with IN10018. The cells except CO‐04‐0070 PDC were treated with different doses of IN10018 for 72 h. (Data represent mean ± SEM, *n* ≥ 3). B–L) In vivo evaluation of IN10018 effects on different cancer models taking KRAS mutation. The models were treated with vehicle control (0.5% Natrosol 250 HX) and 25 or 50 mg kg^‐1^ of IN10018. Body weights and tumor sizes of the mice were monitored twice a week. (Data represent mean ± SEM, *n* ≥ 5). Statistics analysis was done using unpaired student's *T*‐test. *P < 0.05, **P < 0.01, and ***P < 0.001. M–P) Western blot results for FAK signaling upon IN10018 treatment. The tumors from NCI‐H358, NCI‐H2122, SNU668, and mouse KPL models treated with IN10018 were taken for Western blot tests of FAK signaling.

Initial in vitro evaluations showed that IN10018 has moderate to good efficacy against KRAS mutant cancer cell lines (**Figure** **2**A); we confirmed significant inhibition of FAK activity for the examined cell lines (Figure S3A–G, Supporting Information). We next dosed several KRAS mutant cell line derived xenograft (CDX) and patient‐derived xenograft (PDX) models including NSCLC, CRC, Pancreatic cancer, ovarian cancer, and esophageal cancer with IN10018. IN10018 (25 or 50 mg kg^‐1^, p.o., daily) conferred strong tumor growth inhibition effects against multiple CDX and PDX KRAS mutant tumors in vivo (Figure 2B–L). After sacrificing mice at the endpoint, immunoblotting against Phospho FAK Y397 and total FAK in extracts from dissected tumors of 4 in vivo models. FAK signaling activity was obviously decreased for IN10018‐treated tumors (Figure 2M–P). These results provide direct evidence linking KRAS mutation to altered FAK signaling and prove that FAK inhibition by IN10018 is effective to KRAS mutant cancers.

### 2.3 A Combination Therapy of KRAS G12C and FAK Inhibition Exerts Stronger Anti‐KRAS G12C Mutant Cancer Cell Growth Than Either Monotherapy

**Figure 3:**
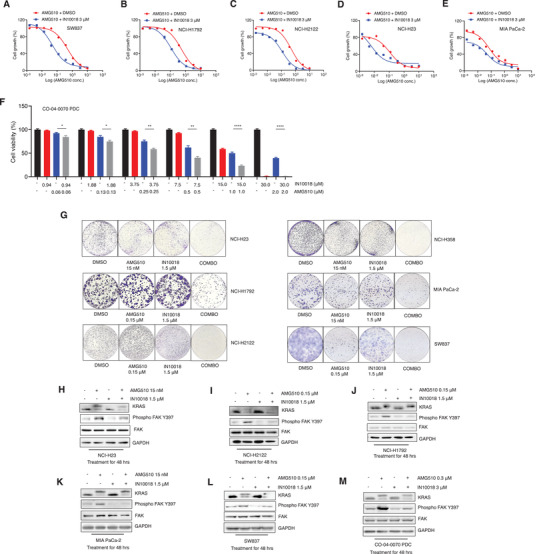
A combination therapy of KRAS G12C and FAK inhibition exerts stronger anti‐KRAS G12C mutant cancer cell growth than either monotherapy. A–E) Drug combination test of IN10018 and AMG510 on 5 KRAS G12C mutant cancer cell lines. The cells were treated with the combination of 3 × 10^‐6^ m IN10018 and serial dilution of 10 × 10^‐6^ m AMG510 for 72 h. CTG assay was performed for cell viability evaluation. (Data represent mean ± SEM, *n* ≥ 3). F) Drug combination test of IN10018 and AMG510 on CO‐04‐0070 PDC. The CO‐04‐0070 PDC cell line was incubated with different concentrations of AMG510 and IN10018 for 120 h. CTG assay was performed for cell viability evaluation. (Data represent Mean ± SEM, *n* = 4). Statistics analysis was unpaired student's *T*‐test. *P < 0.05, **P < 0.01, and ****P < 0.0001. G) Cell clonogenic assay for the combination of AMG510 and IN10018. The cells were treated with AMG510 and IN10018 for 10 d. The cell colonies were stained with 0.1% crystal violet for evaluation. H–M) Western blot tests for FAK signaling after drug combination of AMG510 and IN10018. The cells were treated with test articles for 48 h. Total protein was extracted for Western blot.

The cell viability assay was used to test a combination of AMG510 and IN10018 with diverse KRAS G12C mutant cancer cell lines. For all of the tested cell lines including a CRC cell line (SW837), a Pancreatic cancer cell line (Mia PaCa‐2), a CRC patient‐derived cell line (PDC) (CO‐04‐0070‐PDC), and 3 NSCLC cell lines (NCI‐H23, NCI‐H1792, and NCI‐H2122), the combination treatment conferred stronger cytotoxic effects than KRAS G12C inhibition monotherapy or the FAK inhibition monotherapy (**Figure** **3**A–F; Figure S5E, Supporting Information). Synergistic effects were evaluated using Bliss^[^ ^25^ ^]^ and combination index (CI)^[^ ^26^ ^]^ analysis (Figure S4A,B, Supporting Information). *PTK*2 knockdown by siRNA or FAK inhibition by another 2 small molecule inhibitors Defactinib and GSK2256098 were also applied to confirm the findings from the drug combination tests with AMG510 and IN10018 (Figure S4C‐I, Supporting information). Similar results were observed for a combination therapy comprising MRTX849 and IN10018 for 72 h, assayed against 3 NSCLC cell lines (Figure S5A–D, Supporting Information).

To test the long‐term (10 days) effects of the AMG510 and IN10018 drug combination, we performed cell colony formation assays with diverse KRAS mutant cancer, using specific doses of each combination therapy agent (based on individually testing AMG510 and IN10018 for each cell line). In line with our results from shorter‐term testing (above), the combination therapy consistently outperformed the monotherapies for cancer cell killing (Figure 3G). Similar results were obtained with the long‐term MRTX849‐IN10018 combination therapy against all 4 tested NSCLC cell lines (NCI‐H23, NCI‐H358, NCI‐H1792, and NCI‐H2122) (Figure S5F, Supporting Information). We also monitored FAK signaling activity at the 48 h postadministration time point of the AMG510–IN10018 combination therapy in the various KRAS G12C mutant cell lines, which revealed clear induction of Phospho FAK Y397 after KRAS G12C inhibition. Further, the impact of IN10018 in significantly decreasing FAK activity in the treated cells was evident by the obviously decreased accumulation of Phospho FAK Y397 (Figure 3H–M).

### 2.4 FAK Inhibition Specifically Enhances the Cancer Cell Killing Effects of KRAS G12C Inhibitors by Repressing FAK‐YAP Signaling

**Figure 4:**
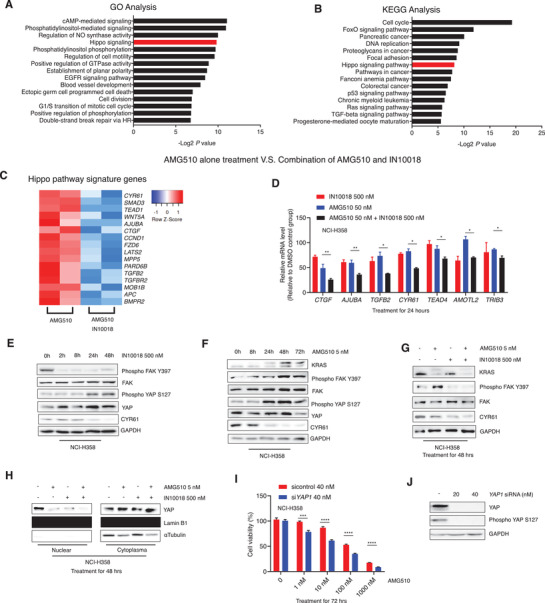
FAK inhibition specifically enhances the cancer cell killing effects of KRAS G12C inhibitors by repressing FAK‐YAP signaling. A) GO analysis for the significantly downregulated genes by the combination of IN10018 and AMG510 compared to AMG510 monotherapy on NCI‐H358 cell line. The Top 15 downregulated KEGG signal pathways were listed here. B) KEGG analysis for the significantly downregulated genes by the combination of IN10018 and AMG510 compared with AMG510 single treatment. The Top 15 downregulated GO signal pathways were listed here. C) Expression levels of Hippo pathway signature genes for the 24 h treatment of AMG510 and the combination of AMG510 and IN10018 to NCI‐H358 cell line. D) Expression levels of Hippo pathway signature genes by RT‐qPCR. The gene expression data of 24 h treated NCI‐H358 cells were normalized to the DMSO control group. (Data represent Mean ± SEM, *n* ≥ 3). Statistics analysis was done using unpaired student's *T*‐test. *P < 0.05, **P < 0.01. E) Western blot tests on NCI‐H358 cells with IN10018 treatment. F) Western blot tests on NCI‐H358 cells with AMG510 treatment. G) Western blot for the combination of AMG510 and IN10018. The biomarkers were detected by Western blot for NCI‐H358 treated with AMG510 alone, IN10018 alone, and the combination of AMG510 and IN10018 for 48 h. H) The detection of Nuclear/Cytoplasm YAP for NCI‐H358 cells treated with the combination of AMG510 and IN10018 for 48 h. I,J) Knockdown of YAP exhibited better cancer cell killing of NCI‐H358 to AMG510. NCI‐H358 cells were transfected with control or *YAP*1 siRNA. 48 h later, AMG510 was dispensed to the cell plates. The cell viability was evaluated 72 h later. Western blot was performed for the knockdown efficiency at the end of the test (Data represent Mean ± SEM, *n* = 4). Statistics analysis was done using unpaired student's *T*‐test. ***P < 0.001, and ****P < 0.0001.

The Hippo pathway is directly regulated by the transcription regulator YAP.^[^ ^27^ ^]^ Previous reports indicated that YAP signaling impacts the development of drug resistance to various cancer therapies.^[^ ^28^, ^29^ ^]^ We conducted RNA‐seq‐based transcriptome profiling of KRAS G12C inhibition sensitive NSCLC (NCI‐H358) cells treated with vehicle control, AMG510, IN10018, or the AMG510–IN10018 combination for 24 h. Briefly, GO and KEGG analyses revealed enrichment for functional annotation relating to the Hippo pathway among the downregulated differentially expressed genes from the combination therapy versus AMG510 monotherapy groups. This finding suggests that FAK inhibition may somehow counteract YAP‐signaling‐mediated compromised antitumor mechanisms related to KRAS G12C inhibition (**Figure** **4**A,B). Further, our transcriptome data showed the cells given the combination therapy had significantly reduced levels of known Hippo pathway signature genes compared to the AMG510 monotherapy cells (Figure 4C).

A similar analysis was also performed for the MRTX849–IN10018 combination, and the consistent results showing Hippo pathway deregulation specifically from dual KRAS G12C and FAK inhibition (Figure S6A,B, Supporting Information), confirming our previous findings. We also used RT‐qPCR to measure the expression of 7 Hippo pathway signature genes in NCI‐H358 cells treated with AMG510, IN10018, or the AMG510–IN10018 combination for 24 h. The combined inhibition of KRAS G12C and FAK caused a significantly greater repression of all the examined Hippo pathway genes compared to the monotherapies (Figure 4D).

We then examined the specific impact of FAK inhibition on YAP signaling via Western blotting of IN10018 treated NCI‐H358 cells. Phospho YAP S127 which is indicative of YAP signaling inhibition,^[^ ^30^ ^]^ was accumulated within 24 h of IN10018 treatment. Consistently, the level of CYR61, a downstream marker of YAP signaling, was apparently reduced by IN10018 treatment (Figure 4E). We also found that for NCI‐H358 cells, AMG510 monotherapy decreased YAP signaling (Figure 4F). When we expanded these immunoblotting analyses to cells with simultaneous inhibition of KRAS G12C and FAK inhibition, we found that the AMG510‐IN10018 combination induced a stronger decrease of CYR61 level compared to either mono‐treatment (Figure 4G). This finding is consistent with our RNA‐seq and qPCR data.

FAK is known to activate and accumulate YAP in cell nucleus through its regulation of the MOB1/LATS1/2 complex, which is an upstream biomarker of YAP signaling^[^ ^31^, ^32^ ^]^ (Figure S7A–F, Supporting Information). We also performed fractionation studies of the variously treated NCI‐H358 cells to examine nuclear and cytoplasmic YAP protein accumulation, and observed that the AMG510 or IN10018 monotherapies caused decreased nuclear YAP accumulation within 48 h. We detected a further decrease of the nuclear YAP level in the drug combination group, indicating a specific enhanced impact on the YAP activity (Figure 4H). Together these results indicating strengthened downregulation of YAP signaling activity upon simultaneous inhibition of KRAS G12C and FAK. Finally, we found that siRNA‐mediated knockdown of YAP expression made NCI‐H358 cells more sensitive to killing upon the AMG510 monotherapy (Figure 4I,J), revealing that the previously demonstrated synergism from FAK inhibition is apparently mediated via YAP signaling.

### 2.5 Downregulation of FAK–YAP axis Potentiates AMG510‐Mediated Cancer Cell Killing in a KRAS G12C Inhibition Resistance Cell Line

**Figure 5:**
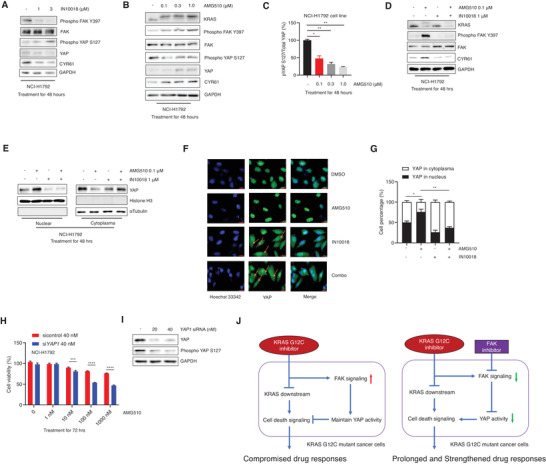
Downregulation of FAK‐YAP axis potentiates AMG510‐mediated cancer cell killing in a KRAS G12C inhibition resistance cell line. A) Western blot for the NCI‐H1792 cells treated with IN10018 for 48 h. The protein samples were extracted for expression levels of Phospho FAK Y397/Total FAK, Phospho YAP S127/Total YAP, and CYR61. B) Western blot for the NCI‐H1792 cells treated with AMG510 for different time points. The protein samples were used to check with Western blot. C) The ratios of phospho YAP S127/total YAP for NCI‐H1792 which was treated with different doses of AMG510 for 48 h. (Data represent mean ± SEM, *n* = 3). Statistics analysis was done using one‐way ANOVA. *P < 0.05, **P < 0.01. D) Western blot for the combination treatment of AMG510 and IN10018 in NCI‐H1792 cells. The NCI‐H1792 cells were treated with inhibitors for 48 h. The protein was used for biomarker detection. E) The detection of nuclear/cytoplasm YAP for NCI‐H1792 cells treated with the combination of AMG510 and IN10018 for 48 h. F) The immunofluorescence test for YAP protein for NCI‐H1792 treated with test articles. The treatment is the same as (E). Scale bar = 20 µm. Yellow arrows indicated the cytoplasm YAP staining. G) The Image J analysis for the YAP staining results from (F). (Data represent Mean ± SEM, *n* = 4). Statistics analysis was done using unpaired student's *T*‐test. *P < 0.05, **P < 0.01. H,I) Knockdown of YAP results in enhanced cell viability inhibition for NCI‐H1792 cells treated with AMG510. The procedure is the same as (Figure 4I,J). (Data represent mean ± SEM, *n* = 4). Statistics analysis was done using unpaired student's *T*‐test. ***P < 0.001, and ****P < 0.0001. J) The schematic diagram summarizing the major findings from the study.

Given the evidence from ongoing clinical trials that a substantial proportion of KRAS G12C mutant cancer patients do not benefit from KRAS inhibition therapy,^[^ ^33^ ^]^ we explored whether the FAK–YAP axis may explain the resistance to KRAS G12C inhibition. Experiments with the AMG510 resistant cell line NCI‐H1792 revealed AMG510 dose‐dependent decreases in the Phospho YAP S127‐total YAP ratio, which is commonly used as a negative marker for YAP signaling^[^ ^34^ ^]^ (**Figure** **5**B,C; Figure S6C,D, Supporting Information). As with our results for NCI‐H358 cells, immunoblotting showed that IN10018 treatment efficiently decreased YAP signaling activity (Figure 5A). Moreover, the AMG510‐IN10018 combination treatment of NCI‐H358 cells caused an apparent reduction in the accumulation of the downstream YAP component CYR61 compared to the AMG510 mono‐treatment (Figure 5D). Western blot‐based and Immunofluorescence‐based monitoring of nuclear YAP level showed that AMG510 treatment promotes nuclear accumulation, whereas the AMG510‐IN10018 combination treatment caused a net reduction in the YAP accumulation compared to AMG510 alone (Figure 5E–G). Further, *YAP*1 siRNA also exhibited a strengthened effect to NCI‐H1792 cell killing by AMG510 (Figure 5H,I). Finally, LATS1/LATS2 knockdown can rescue the cancer cell killing effect from the combination treatment of AMG510 and IN10018, suggesting Hippo kinase signaling is involved in this FAK‐YAP related drug response (Figure S6E,F, Supporting Information). Our data showed that both KRAS G12C inhibition sensitive and resistant cell lines benefited from the combination of KRAS G12C and FAK inhibition through the regulation of the FAK‐YAP axis. A schematic diagram mechanistically elucidating the findings is shown here (Figure 5J).

### 2.6 The Combination of KRAS G12C and FAK Inhibition Exerts Synergistic Effects Against CDX Models of Pancreatic and NSCLC Harboring KRAS G12C Mutation

**Figure 6:**
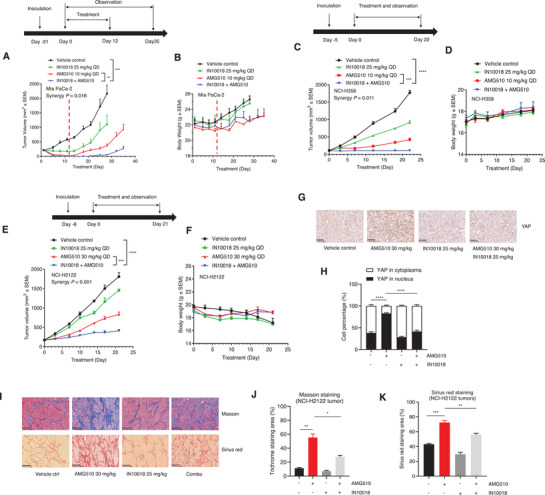
The combination of KRAS G12C and FAK inhibition exerts synergistic effects against CDX models of pancreatic and NSCLC harboring KRAS G12C mutation. A,B) The tumor growth and body weight changes of Mia PaCa‐2 xenografts treated with the indicated agents. The dosing was stopped on the 12th day due to regression of all the tumors in the combination group. (Data represent mean ± SEM, *n* = 4, synergy *P* value is shown). Statistics was done unpaired student's *T*‐test for the tumor volume data at day 28 of the study. **P < 0.01, and ***P < 0.001. C,D) The tumor growth and body weight changes of NCI‐H358 xenografts treated with the test articles. (Data represent mean ± SEM, *n* = 5, Synergy *P* value is shown). Statistics was done using unpaired student's *T*‐test at the end of the study. ***P < 0.001, and ****P < 0.0001. E,F) The tumor growth and body weight changes of NCI‐H2122 xenografts treated with the indicated agents. (Data represent mean ± SEM, *n* = 5, synergy *P* value is shown). Statistics was performed unpaired student's *T*‐test at the end of the study. ***P < 0.001, and ****P < 0.0001. G) The IHC staining of YAP protein for the tumors from the NCI‐H2122 study. Scale bar = 50 µm. H) The Image J analysis for the YAP IHC results from (G). (Data represent Mean ± SEM, *n* = 4). Statistics analysis was done using unpaired student's *T*‐test. ****P < 0.0001. I) Masson and Sirius red staining for the NCI‐H2122 tumors. Scale bar = 100 µm. J) The Image J analysis of Masson staining of (I). K) The Image J analysis of Sirius red staining of (I). (Data represent mean ± SEM, *n* = 3). Statistics was done using unpaired student's *T*‐test. *P < 0.05, **P < 0.01, and ***P < 0.001.

Experiments with two KRAS G12C inhibition‐sensitive CDX models Mia PaCa‐2 (Pancreatic cancer) and NCI‐H358 (NSCLC) were used to evaluate the potential therapeutic utility of the AMG510‐IN10018 combination. These experiments included both monotherapies, and synergistic effects on tumor growth inhibition were observed for the combination group (25 mg kg^‐1^ IN10018 and 10 mg kg^‐1^ AMG510). Note that tumor regression was observed with Mia PaCa‐2 model animals for both the combination therapy and AMG510 monotherapy groups. The dosing was stopped at treatment day 12; later, the regrowth of tumors was obviously delayed in the combination therapy compared to the AMG510 monotherapy group. With the NCI‐H358 model, the AMG510‐IN10018 combination outperformed both of the monotherapies in terms of tumor growth inhibition. The synergy *P* values were calculated to represent the synergistic effects for all the following in vivo studies.^[^ ^35^ ^]^ Note that no obvious body weight decreases were observed upon treatment for either of the two models, indicating good tolerability (**Figure** **6**A–D).

We also established a KRAS G12C inhibition resistant NSCLC CDX model (NCI‐H2122 cell line) to support in vivo testing. We found that IN10018 (25 mg kg^‐1^) synergizes with AMG510 (30 mg kg^‐1^) for tumor growth inhibition, again without any abnormalities (Figure 6E,F). Experimentally reinforcing our findings from the mechanistic studies with NCI‐H1792 cells, we observed that the AMG510 treated NCI‐H2122 tumors displayed elevated YAP signaling activity, as assessed via YAP IHC (Figure 6G,H), as the Phospho YAP S127: total YAP ratio, Phospho YAP S127: GAPDH, and YAP: GAPDH (Figure S8A‐D, Supporting Information), and as the YAP downstream CYR61 IHC (Figure S8E,F, Supporting Information). As anticipated from our earlier in vitro work, we observed that the AMG510 treatment efficiently reduced this ratio, whereas simultaneous FAK inhibition rescued the ratio, and the presence of IN10018 in the treatment reduced the extent of nuclear YAP induced by AMG510.

Feng and co‐workers found that FAK inhibition not only affects YAP activity but also interferes with YAP stability.^[^ ^31^ ^]^ Our findings in the NCI‐H2122 model reflect this: long‐term treatment including IN10018 caused an overall decreased in total YAP levels (Figure S8A, Supporting Information).

Interestingly, Masson and Sirius red staining of dissected NCI‐H2122 tumors indicated that long‐term AMG510 treatment at 30 mg kg^‐1^ dose resulted in more fibrogenesis compared to the vehicle control tumors (Figure 6I–K). Previous studies have reported that FAK inhibition can decrease fibrogenesis‐related aberrant stromal proliferation,^[^ ^31^, ^36^ ^]^ consistently we found that FAK inhibition by IN10018 also markedly decreased the extent of high‐dose AMG510 related fibrosis. Since excessive fibrosis also acts as the obstacle of obtaining anticancer effects from drug treatments,^[^ ^37^ ^]^ this finding provides more evidence for the benefit from the drug combination of KRAS G12C and FAK inhibition.

### 2.7 The Combination of KRAS G12C and FAK Inhibition Exhibits Synergistic Effects in KRAS G12C Mutant PDX Models

**Figure 7:**
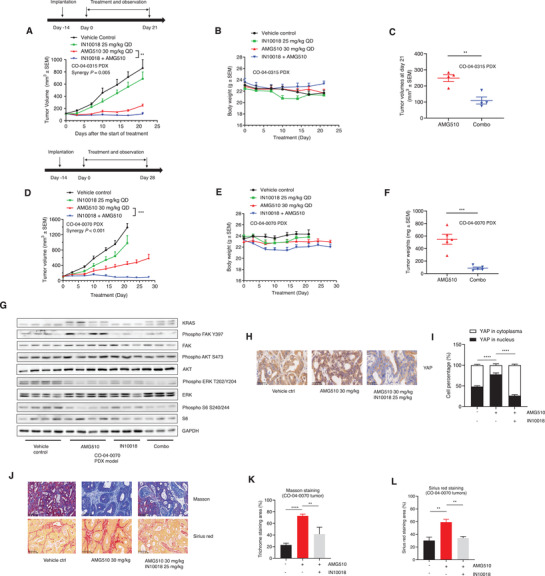
The combination of KRAS G12C and FAK inhibition exerts synergistic effects against PDX models from KRAS G12C mutant CRC patients. A,B) Tumor growth and body weight changes of CO‐04‐0315 PDX models treated with vehicle control (0.5% Natrosol 250 HX), 30 mg kg^‐1^ AMG510, 25 mg kg^‐1^ IN10018, and the combination of AMG510 and IN10018. (Data represent Mean ± SEM, *n* = 4, synergy *P* value is shown). Statistics analysis was done using unpaired student's *T*‐test for the tumor volume data at the end of the study. **P < 0.01. C) Tumor volumes of CO‐04‐0315 PDX models at day 21 post drug treatment. (Data represent mean ± SEM, *n* = 4). Statistics analysis was done using unpaired student's *T*‐test. **P < 0.01. D,E) Tumor growth and body weight changes of CO‐04‐0070 PDX models treated with the indicated agents. (Data represent mean ± SEM, *n* ≥ 4, synergy *P* value is shown). Statistics was performed using unpaired student's *T*‐test at the end of the study. ***P < 0.001. F) Tumor weights from AMG510 single and combination groups of CO‐04‐0070 PDX models. (Data represent mean ± SEM, *n* = 5). Statistics analysis was done using unpaired student's *T*‐test. ***P < 0.001. G) Western blot tests for the tumors of CO‐04‐0070 models. H) The IHC staining of YAP protein of CO‐04‐0070 tumors. Scale bar = 50 µm. I) The analysis for the YAP IHC results from (H). Image J was used for YAP IHC analysis. (Data represent Mean ± SEM, *n* = 4). Statistics analysis was done using unpaired student's *T*‐test. ****P < 0.0001. J) Masson and Sirius red staining for the tumors of CO‐04‐0070 models. Scale bar = 100 µm. K) The image J analysis for the Masson staining of (J). L) The image J analysis for the Sirius red staining of (J). (Data represent Mean ± SEM, *n* = 4). Statistics analysis was done using unpaired student's *T*‐test. **P < 0.01, ****P < 0.0001.

Given that KRAS G12C inhibitors did not result in good response rates for colorectal cancer patients in clinical trials,^[^ ^33^ ^]^ we conducted in vivo evaluation of the combined KRAS G12C and FAK inhibition therapy using two CRC PDX models: CO‐04‐0315 and CO‐04‐0070. CO‐04‐0315 is relatively sensitive to 30 mg kg^‐1^ AMG510 and the combination of AMG510 and 25 mg kg^‐1^ IN10018 exhibited better anti‐tumor effects compared to either of the monotherapies (**Figure** **7**A,C). The mice tolerated the treatments well during the drug dosing period (Figure 7B).

For the PDX model CO‐04‐0070, both the vehicle control and 25 mg kg^‐1^ IN10018 groups were taken down on day 21 because of excessive tumor burdens. The KRAS G12C inhibition group (30 mg kg^‐1^ AMG510) showed persistent tumor growth in these experiments, highlighting the impact of the drug resistance to AMG510 of the CO‐04‐0070 model. Unlike either monotherapy, the AMG510‐IN10018 combination conferred tumor regression (Figure 7D). During the dosing period, all of the animals tolerated the treatments well (Figure 7E). Analysis of dissected tumor weights showed that the combination group tumors were significantly smaller than the AMG510 monotherapy group providing another line of evidence for the treatment benefits from the combination therapy (Figure 7F; Figure S9K, Supporting Information). Western blotting and pathology experiments showed that the AMG510 group tumors exhibited apparent inhibition of FAK activity, and the presence of IN10018 in the combination group efficiently reduced this activity (Figure 7G).

Similar to the NCI‐H2122 animal study, AMG510 treatment resulted in significantly more nuclear YAP accumulation compared to the vehicle control group in CO‐04‐0070 model and the combination of AMG510 and IN10018 efficiently reduced nuclear YAP accumulation (Figure 7H,I). Masson and Sirius red staining indicated that excessive fibrosis was generated by AMG510 treatment, the inhibition of FAK by IN10018 sharply decreased the fibrosis (Figure 7J–L).

We also employed a KRAS G12C mutant NSCLC PDX model LU‐01‐0030 which displays primary resistance to KRAS G12C inhibition for additional drug evaluation. The initial dose of AMG510 was set to 10 mg kg^‐1^: neither AMG510 alone nor in combination with 25 mg kg^‐1^ of IN10018 led to apparent tumor growth inhibition. Thus, at 21 d after starting treatment, the dose of AMG510 was increased to 30 mg kg^‐1^. The combination group showed better anti‐cancer effects compared to AMG510 alone treatment without adverse effects (Figure S9A–C, Supporting Information). FAK‐YAP axis activation and fibrogenesis were also confirmed by Western blotting and pathology experiments in this model (Figure S9D–J, Supporting Information).

## 3 Discussion

KRAS mutations were considered undruggable until the emergence of KRAS G12C inhibitors.^[^ ^38^ ^]^ Promising preclinical and clinical results have subsequently boosted the development of inhibitors targeting the KRAS G12C mutation.^[^ ^21^, ^39^ ^]^ To date, two pioneer KRAS G12C inhibitors (AMG510 and MRTX849) have entered phase 2 clinical trials for the treatment of cancers harboring KRAS G12C mutations as a single drug or in combination with other therapies.^[^ ^40^ ^]^ Preclinical and clinical data have revealed that drug resistance to KRAS G12C inhibitors eventually develops with long‐term treatment.^[^ ^6^, ^33^, ^41^ ^]^ Recent studies have shown that strategies to combine the inhibitors targeting either KRAS upstream or downstream signaling components with KRAS G12C inhibitors can prolong the antitumor effects of treatments, indicating that the vertical signaling of KRAS is important to maintain the drug effects.^[^ ^8^, ^9^, ^20^ ^]^ The evidence clearly emphasizes that modulating targets adjacent to KRAS provides possibilities for overcoming the resistance‐development‐related compromised drug effects from KRAS G12C inhibitors.

FAK captured and retained our interest in this study because of several unique attributes. First, FAK is a downstream signaling component of KRAS, emphasizing its directly linked function in supporting the KRAS activity.^[^ ^14^, ^42^ ^]^ Second, FAK inhibitors have been reported to exert preferential killing to KRAS mutant cancer.^[^ ^12^ ^]^ Third, released data from a clinical trial indicated that good anti‐cancer responses can be achieved for KRAS mutant cancers based on FAK inhibition (NCT03875820).^[^ ^43^ ^]^ We focused on a clinical‐stage FAK inhibitor: IN10018, which was previously developed by Boehringer‐Ingelheim under the name BI853520. Our data suggested that IN10018 exerts therapeutic effects on multiple KRAS mutant cancer models, providing us a solid empirical basis and set of models for elucidating the biological connections between FAK signaling and KRAS activity. Interestingly, multiple previous studies have implicated FAK activation in the development of resistance against various chemotherapy agents through its regulation of the tumor microenvironment.^[^ ^11^, ^15^, ^44^ ^]^ FAK signaling is hyperactivated by long‐term treatments that target KRAS vertical signaling components (e.g., RAF and EGFR).^[^ ^45^ ^]^

Similar to the function of FAK activation within other drug resistance contexts, in the present study we found that KRAS G12C inhibition (by either AMG510 or MRTX849) significantly stimulates FAK activity, providing a rationale for the combination treatment of KRAS G12C and FAK inhibition. We observed synergistic effects for such drug combinations against cancer cell lines as well as CDX, and PDX models harboring the KRAS G12C mutation. Mechanistically, we demonstrate that abnormal FAK–YAP axis signaling account for drug‐resistance‐related outcomes of KRAS G12C inhibition. On the one hand, it is known that YAP activity is positively correlated with the resistance to therapies targeting KRAS signaling components including RAF and MEK,^[^ ^28^, ^46^ ^]^ and YAP is required for KRAS‐dependent cell transformation: extracellular supplementation of YAP activators can rescue cancer cell killing after KRAS knockdown.^[^ ^47^ ^]^ On the other hand, FAK was reported to positively regulate YAP activity through tyrosine phosphorylation of the YAP upstream modulator MOB1, then dissociating the MOB1/LATS complex.^[^ ^31^, ^32^ ^]^ Our data from transcriptome and protein analyses showed that combination treatment against KRAS G12C and FAK inhibition caused a stronger reduction in YAP activity than either monotherapy. Viewing these lines of evidence from other studies and our own data together, we hypothesized that aberrantly strong FAK–YAP signaling can plausibly explain the development of resistance against the long‐term use of KRAS G12C inhibitors.

Further evidence from experiments with a KRAS G12C inhibition sensitive NSCLC cell line (NCI‐H358) showed that the combination treatment comprising AMG510 and IN10018 can induce more substantial decrease in YAP activity than either monotherapy. Further, our data from experiments with multiple primary KRAS G12C inhibition‐resistant cancer models showed elevated YAP activity upon AMG510 treatment and significant reductions in the extent of YAP activation upon the combination treatment (AMG510 and IN10018). We also detected that enhanced effects from AMG510 for cancer cell killing result from either IN10018 or *YAP*1 siRNA, further supporting that the aberrantly activated FAK–YAP signaling contributes to the compromised efficacy of KRAS G12C inhibitors. While our data do support that YAP inhibitors may also exert synergistic effects to KRAS G12C inhibition, a capacity for small‐molecule‐mediated direct targeting of YAP remains elusive.^[^ ^27^ ^]^ Thus, FAK inhibition can substitute for direct YAP inhibition to maximize the long‐term anti‐cancer outcomes from use of KRAS G12C inhibitors.

FAK signaling has been reported to regulate fibrogenesis within tumors,^[^ ^17^, ^48^ ^]^ and excessive fibrosis is correlated with drug resistance, because it can create a barrier that prevents interactions between cancer cells and therapeutic agents.^[^ ^49^ ^]^ In the present study, AMG510 treatment of three models (1 NSCLC CDX (NCI‐H2122), 1 CRC PDX (CO‐04‐0070), and 1 NSCLC PDX (LU‐01‐0030)) resulted in excessive fibrogenesis, and this was accompanied in each case by hyperactivated FAK signaling. The combination treatment of AMG510 and IN10018 significantly decreased the extent of fibrosis in these tumors, results both supporting that FAK‐related fibrosis may also limit the anticancer effects of KRAS G12C inhibitors and highlighting an additional mechanism through which the combination therapy we tested can help preclude development of drug resistance.

A recent report revealed that immunotherapy efficacy can be enhanced by KRAS G12C inhibition, owing specially to the resulting enhancement of tumor infiltration of CD8^+^ T cells, macrophages, and dendritic cells.^[^ ^21^ ^]^ FAK‐related fibrosis can create a barrier in tumors that limits the tumor infiltration of CD8^+^ T cells,^[^ ^17^ ^]^ and it is known that the FAK inhibition can decrease the number of tumor‐resident Tregs, ultimately promoting CD8^+^ T cell‐regulated antitumor effects,^[^ ^50^ ^]^ our findings suggest that FAK inhibition may facilitate the combination strategy of immunotherapy and KRAS G12C inhibition which is being tested on a clinical trial (NCT04185883). A triplet strategy may thus further enhance CD8^+^ T cell infiltration within tumors to benefit immunotherapy‐related outcomes. There is reported evidence showing combined benefits from a PD1 antibody in combination with FAK inhibition against KRAS mutant cancer.^[^ ^17^ ^]^ So, we strongly suspect that a triple combination of KRAS G12C, FAK, and PD1 inhibitors may provide further benefits to treatment outcomes against KRAS G12C mutant cancers.

In summary, FAK signaling is hyperactivated by KRAS G12C inhibition, inducing compromised treatment outcomes via dysregulated FAK‐YAP signaling and through fibrosis formation. Drug combinations comprising KRAS G12C inhibitors and the FAK inhibitor IN10018 showed promising synergistic effects against diverse cancer cells and multiple cancer models, including NSCLC, CRC, and pancreatic cancer. Given the prevalence of the KRAS G12C mutation in diverse malignancies, it seems that this (and related) combination strategies can benefit the treatment outcomes of many cancer patients. Based on our data, planning for a clinical trial is underway: it will examine the combination treatment of KRAS G12C inhibitors and IN10018 for KRAS G12C mutant colorectal cancers soon.

## 4 Experimental Section

### Cell Lines and Reagents

NCI‐H1792, NCI‐H2122, NCI‐H23, NCI‐H358, HCC827, A549, SW1573, SW837, Mia PaCa‐2, TOV‐21G, MDA‐MB‐231, and CALU‐6 cell lines were purchased from American Type Culture Collection (ATCC). KYSE‐410 cells were from the European Collection of Cell Cultures (ECACC). SNU668 and HCC‐44 cells were from the Korean Cell Line Bank (KCLB). CO‐04‐0070 PDC cells were provided by WuXi AppTec. Mouse KPL cell line was a gift of Ji et al.^[^ ^51^ ^]^ Cells were maintained in RPMI1640 or DMEM (Basalmedia) supplemented with 10% fetal bovine serum (FBS) (Gibco) and 1% penicillin–streptomycin (Gibco). AMG510, MRTX849, Defactinib, and GSK2256098 were purchased from DC chemicals. IN10018 was provided by InxMed. All the siRNAs used in the study were synthesized by Genepharma. Primers used in this study were synthesized by Biosune and listed together with siRNA sequences in Table S1, Supporting Information. The antibodies tested in the study were summarized in Table S2, Supporting Information.

### Cell Viability Assay

CellTiter‐Glo cell viability kit was purchased from Promega. The procedures were per protocol. Briefly, substrate and buffer from CellTiter‐Glo kit were mixed to be CellTiter‐Glo reagent. The reagent was dispensed to the wells from 96 well plates which were set for cell viability tests. 10 min later, the plates were transferred to the varioskan flash plate reader (Thermo Fisher Scientific) for data reading. The data were analyzed by Graphpad 8.0. Cell clonogenic assay was performed for long‐term treatment on the cell lines. Briefly, the cells were plated to 12 well plates with 2000 cells per well. On the second day, the treatment agents were added to the plates and the cells were maintained for 10 d with drug treatments. Then, the medium was discarded. The cells were fixed with 4% paraformaldehyde (Sigma‐Aldrich) for 15 min and then stained with 0.1% crystal violet (Sigma‐Aldrich) for another 15 min. Tap water was used for washing out the excess dye from cell plates.

### Western Blot

The protein samples were extracted from cell lines or animal tissues with RIPA lysis buffer (Rockland). The quantitation of protein amounts was performed with the BCA kit (Thermo Fisher Scientific). Then the samples were mixed with 4× laemmli blue loading buffer (Bio‐Rad) for electrophoresis. After transferring, the samples were incubated with primary antibodies at 4 °C overnight. The secondary antibodies were used for 1 h incubation at room temperature. The blotting membranes were excited by ECL reagent (Bio‐Rad) and the exposure was procedured with ChemiDoc MP (Bio‐Rad). Western blot results were analyzed by Image lab software (Bio‐Rad). For the antibody information used in the study please refer to the Table S2, Supporting Information.

### Reverse Transcription and Quantitative Real‐Time PCR (RT‐qPCR)

Total RNA of cell samples was extracted by RNA iso plus reagent (Takara). The reverse transcription was performed with high‐capacity cDNA reverse transcription Kit (Thermo Fisher Scientific). Different gene expressions were tested with corresponding primers on 7500 real‐time PCR (ABI) by iTaq Universal SYBR Green Supermix (Bio‐Rad). Relative expression levels were calculated by the comparative Ct approach. For the primers used in the study please refer to the Table S1, Supporting Information.

### RNA‐Seq

Total RNA was extracted following the procedures of RNA iso plus reagent (Takara). RNA integrity values were detected by RNA 6000 nano kit on 2100 bioanalyzer (Agilent). The cDNA libraries were generated with NEBNext Ultra Directional RNA Library Prep Kit for Illumina (New England Biolabs). Hiseq 2500 sequencer (Illumina) was employed for the sequencing. Raw data were processed with TopHat and Cuffdiff packages.^[^ ^52^ ^]^ The significantly regulated genes were uploaded to DAVID for KEGG and GO signal pathway enrichments.^[^ ^53^ ^]^ Heatmaps were produced by the Heatmapper package.^[^ ^54^ ^]^

### siRNA Transfection

The cell lines were transfected with siRNAs using lipofectamine RNAi MAX reagent (Thermo Fisher Scientific). Briefly, the siRNAs were incubated with lipofectamine containing Opti‐MEM medium (Gibco) for 20 min. Then, the siRNA matrix was added to the cell plates and incubated with cells for the set time. At the end of the experiments, relative protein levels were checked by Western blot to confirm the transfection efficiency.

### Cell Nuclear/Cytoplasm Protein Extraction

The cell nuclear/cytoplasm protein extraction kit was purchased from Beyotime. All the procedures followed the guidelines from the protocol. Protein samples from the nuclear/cytoplasm part were quantitated with BCA kit and the protein expression levels were checked with Western blot.

### Immunofluorescence Assay

The cells were fixed with 4% paraformaldehyde (Sigma‐Aldrich) for 15 min. 0.1% of Triton X was used for the permeation of the cells. The cells were blocked by 3% BSA for at least 1 h at RT. Then, the cells were incubated with YAP primary antibody at 4 °C overnight. On the second day, the cells were incubated with Hoechst 33342 in combination with fluorescence secondary antibody at RT for at least 1 h. The cell containing slides were sealed with fluorescence mounting medium (DAKO) and covered by coverslips. The slides were scanned with fluorescence scanner (Leica).

### TCGA Analysis

The patient survival data and *z* scores for *PTK*2 RNA expression of all the TCGA samples were downloaded from cBioPortal website.^[^ ^19^ ^]^ The z scores less than ‐1 or bigger than +1 were considered as low or high expression criteria. First, the data were separated into 2 cohorts based on the KRAS genotypes of the patients. Then, the data were further separated by z scores of *PTK*2 RNA expression levels. Survival analysis was done by Graphpad 8.0. Log‐rank test was performed for the statistical analysis.

### Mouse Studies

The animal experiment designed in this study was approved by the ethical committee of Shanghai Jiao Tong University School of Medicine (SJTU‐SM). All the animal studies were performed following the AAALAC guidance. In detail, animal experiments for NCI‐H2122, NCI‐H358, SNU668, MDA‐MB‐231, and Mouse KPL models were approved by the Institutional Animal Care and Use Committee (IACUC) of Shanghai Sixin. The animal experiments for TOV‐21G, CALU‐6, and KYSE‐41G were approved by the IACUC of Boehringer‐Ingelheim. The animal experiment for Mia PaCa‐2 was approved by the IACUC of Shanghai Chempartner. The PDX study for NSCLC model LXFL 1674 was approved by the IACUC of Oncotest. The study of the ovarian cancer PDX model CTG‐0964 was approved by the IACUC of Champions and the study for the NSCLC PDX model LU‐01‐0030, CRC PDX models CO‐04‐0070 and CO‐04‐0315 were approved by the IACUC of Shanghai WuXi AppTec. The animal models were generated on BALB/c nude mice and NOD SCID mice. All the test articles were dosed through oral gavage once daily. The vehicle control reagent for the compounds was 0.5% Natrosol 250 HX in distilled water. The body weights and tumor volumes were monitored and recorded twice a week. The tumor volumes were measured by caliper and calculated with a formula of 0.5 × long diameter × short diameter × short diameter. Dosing was initiated when the average tumor volume reached between 100 mm^3^ and 200 mm^3^. The animals were euthanized if tumor sizes were bigger than 2000 mm^3^. The tumors from NCI‐H2122, CO‐04‐0070, and LU‐01‐0030 models were harvested for immunohistochemistry and Western blot.

### Pathological Processing and Immunohistochemistry

The tumor samples were fixed with 4% paraformaldehyde (Sigma‐Aldrich) and processed into formalin‐fixed paraffin‐embedded (FFPE) blocks. The sample slides were prepared for Masson and Sirius red staining. YAP antibody was used for staining with tumors to recognize YAP position within cancer cells. CYR61 antibody was used for staining NCI‐H2122 tumors. KF‐PRO‐120 scanner (KFBIO) was used for scanning the pathology slides. The scanned results were analyzed by Image J software for the YAP protein location.

### Statistical Analysis

All the in vitro experiments were tested at least in triplicate. The in vivo tests were replicated at least for four times. Means ± SEM was used for representing each data point on the displayed figures. Unpaired, 2‐tailed student's *T*‐test was performed to compare the statistical significance between tested and control groups. One‐way ANOVA with Dunnett's method was tested for comparisons of multiple groups. TCGA data were downloaded from cBioPortal website and the log‐rank test was used for comparison of survival outcomes with Kaplan–Meier method. Correlations between different parameters were analyzed using slope coefficient test. The synergy effects for in vitro assays were represented by Bliss scores and combination index (CI) values evaluated through synergy finder 2.0^[^ ^25^ ^]^ and CompuSyn package.^[^ ^26^ ^]^ The synergy *P* values were processed for the synergistic effects of in vivo studies.^[^ ^35^ ^]^ During the whole study, *P* values less than 0.05 were considered to be significant, *P < 0.05, **P < 0.01, ***P < 0.001, ****P < 0.0001. All the statistical analysis was performed with Graphpad 8.0.

## Conflict of Interest

The authors declare no conflict of interest.

## Author Contributions

B.Y.Z., Z.Q.W., and R.B.R. designed the experiment, analyzed data, and wrote the paper. B.Y.Z. and Y.Z. performed the experiments and analyzed data. J.W.Z. helped to develop the methodology and review the paper. P.L. and B.J. helped to perform the animal experiments. Z.Q.W. and R.B.R. conducted the study supervision. The author(s) read and approved the final manuscript.

## Supporting information

Supporting Information

## Data Availability

The data that support the findings of this study are available in PanCancer studies at https://www.cbioportal.org/. These data were derived from the following resources available in the public domain: https://portal.gdc.cancer.gov/
