# Supplementary material for: Focal Adhesion Kinase (FAK) Inhibition Synergizes with KRAS G12C Inhibitors in Treating Cancer through the Regulation of the FAK–YAP Signaling
Source: Adv Sci (Weinh). 2021 Jun 20;8(16):2100250. doi: 10.1002/advs.202100250 (PMC8373085; doi:10.1002/advs.202100250)
Supplement: Supplementary file 1 — Supporting Information [file ADVS-8-2100250-s001.pdf]

WILEY-VCH

© 2021 Wiley-VCH GmbH

ADVANCED SCIENCE
Open Access

Supporting Information

for *Adv. Sci.*, DOI: 10.1002/advs.202100250

Focal adhesion kinase (FAK) inhibition synergizes with KRAS G12C inhibitors in treating cancer through the regulation of the FAK-YAP signaling

Baoyuan Zhang1, Yan Zhang1, Jiangwei Zhang2, Ping Liu1, Bo Jiao1, Zaiqi Wang2, Ruibao Ren1**

Supporting Information

Focal adhesion kinase (FAK) inhibition synergizes with KRAS G12C inhibitors in treating cancer through the regulation of the FAK-YAP signaling

Baoyuan Zhang, Yan Zhang, Jiangwei Zhang, Ping Liu, Bo Jiao, Zaiqi Wang, Ruibao Ren**

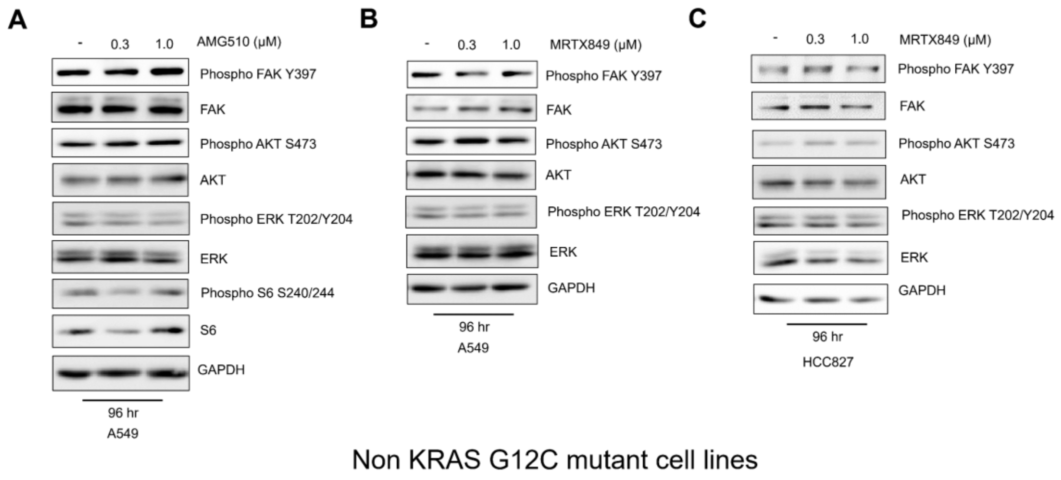

Figure S1. Western blot results for non-KRAS G12C mutant cancer cell lines after KRAS G12C inhibition. A, B) Western blot of KRAS downstream biomarkers of A549 cell line which was treated with AMG510 A) or MRTX849 B) for 96 hours. C) Western blot of KRAS downstream biomarkers of HCC827 cell line which was treated with MRTX849 for 96 hours.

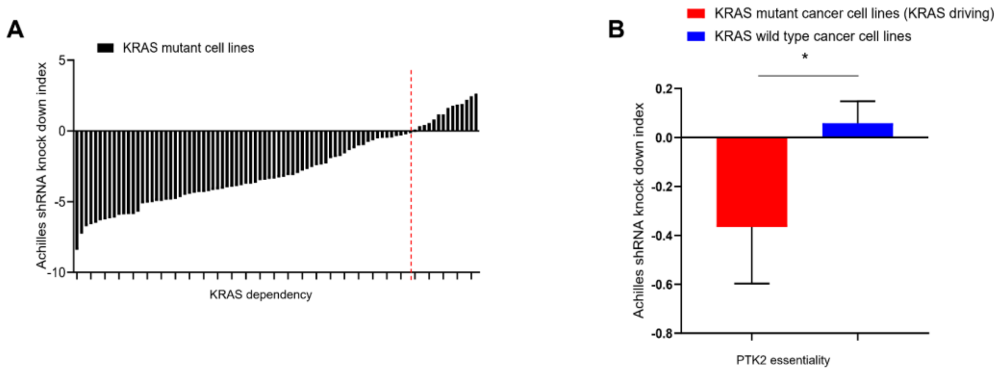

Figure S2. Achilles shRNA screening indicated that FAK knockdown shows preferential killing to the cancer cell lines which show dependence to KRAS mutation. A) The Achilles shRNA knockdown index from cancer cell lines which harbor KRAS mutation. The cell lines showing lower than "0" index were considered as KRAS dependent cancer cell lines. B) Comparison of the *PTK2* shRNA knockdown index for KRAS mutation dependency cell lines and KRAS wildtype cell lines. (Data represent Mean ± SEM, KRAS mutant driving cell lines, n = 71; KRAS wild type cell lines, n = 415). Statistics analysis was done using unpaired student's T test. *$P < 0.05$.

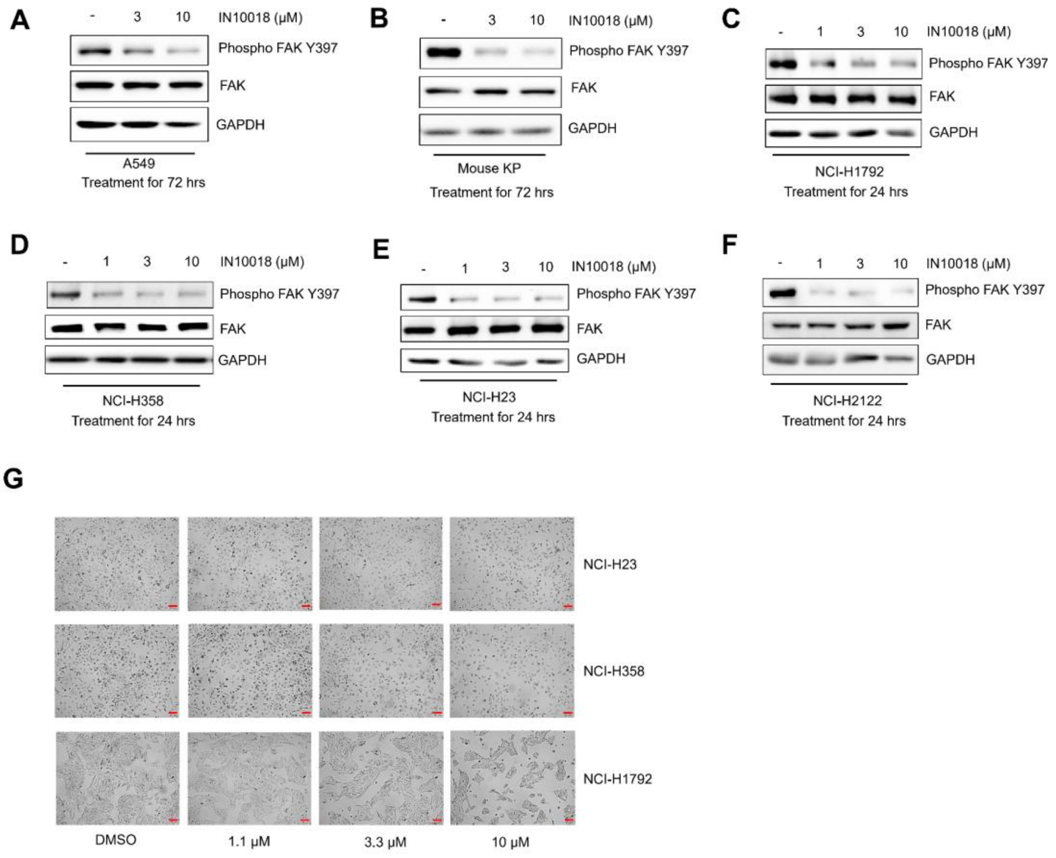

Figure S3. FAK inhibitor IN10018 showed efficacy and suppression of FAK signaling *in vitro*. A-F) The immunoblot of FAK signaling upon the treatment of IN10018 for A549 (A), Mouse KP (B), NCI-H1792 (C), NCI-H358 (D), NCI-H23 (E), and NCI-H2122 (F) cell lines. G) Cell images from 72 hour-cell viability assay of IN10018 for different cell lines. Scale bar = 100 μm.

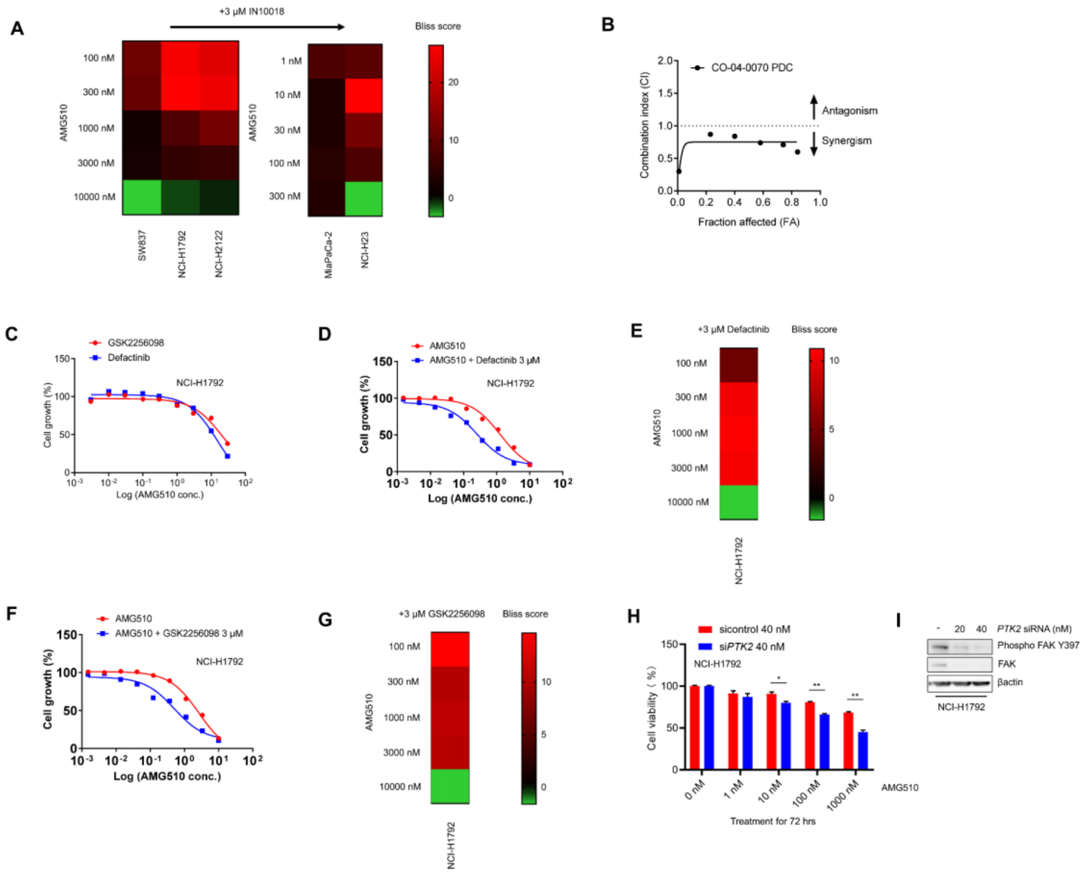

Figure S4. Synergistic analysis of KRAS G12C and FAK inhibition for the *in vitro* tests. A) Bliss analysis for Figure. 3 A-E. Synergy finder 2.0 software was used for Bliss analysis. Bliss score > 0 suggests synergistic effects for the drug combination. B) Combination index (CI) analysis for Figure. 3 F. Compusyn software was used to calculate the CI value. CI value < 0 indicates synergism, CI value = 0 indicates additive effect, and CI value > 0 indicates antagonism. C) Cell viability analysis for NCI-H1792 cell line which were treated with another 2 small molecule FAK inhibitors Defactinib and GSK2256098. D-E) Drug combination analysis for NCI-H1792 which were treated with AMG510 and Defactinib. The synergy analysis was performed using synergy finder 2.0 like (A). F-G) Drug combination analysis for NCI-H1792 which were treated with AMG510 and GSK2256098. The synergy analysis was performed using synergy finder 2.0 like (A). H) Cell viability test for NCI-H1792 cell line treated with combination of *PTK2* siRNA and AMG510. The knockdown and treatment procedures are the same as (Figure 4I and J) (Data represent Mean ± SEM, n = 4). Statistics analysis was done using unpaired student's T-test. $*P < 0.05$, $**P < 0.01$.

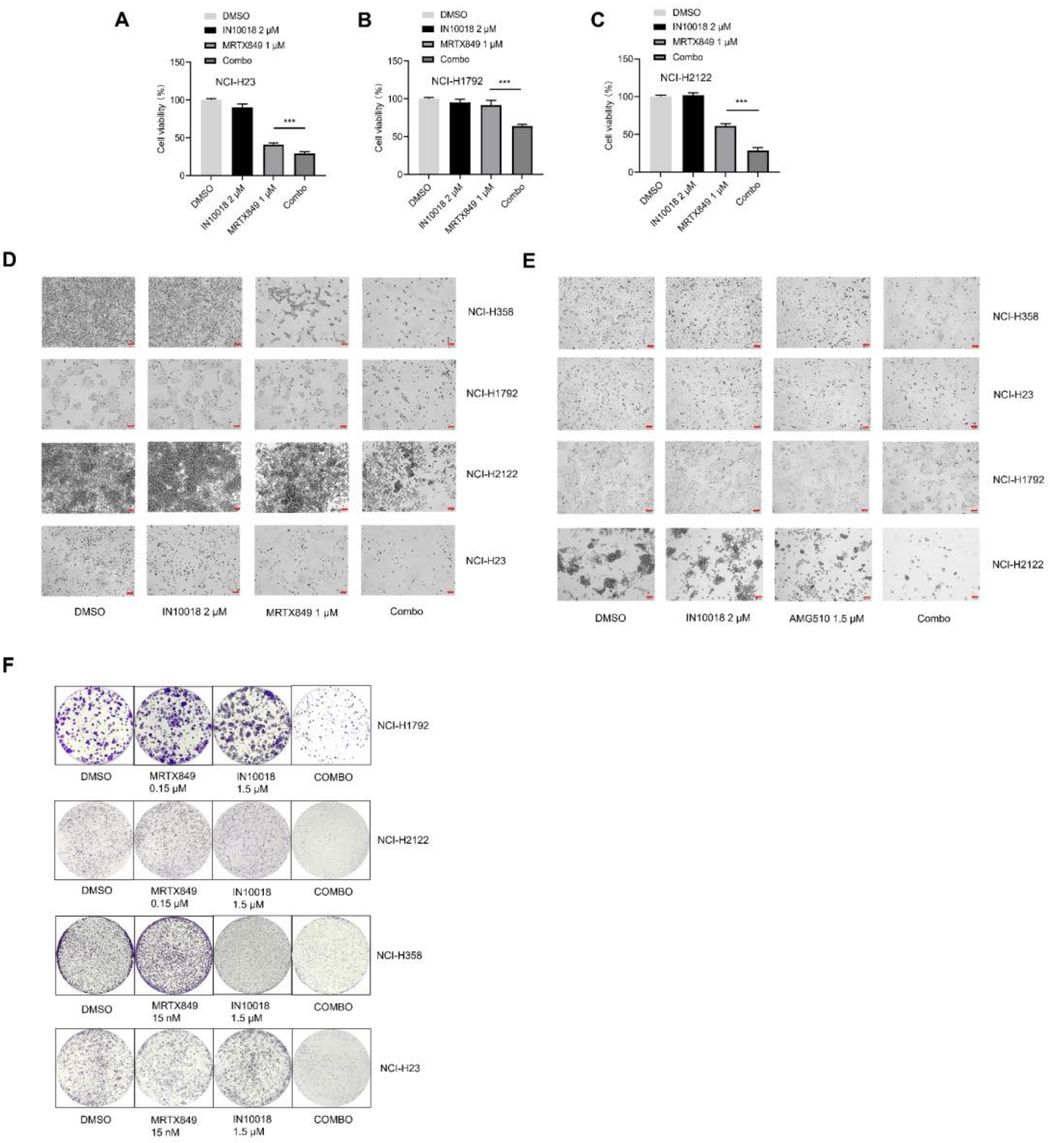

Figure S5. The combination of KRAS G12C inhibition and IN10018 showed synergistic effects to KRAS G12C mutant cancer cell lines. A-C) Drug combination test of NCI-H23 A), NCI-H1792 B), and NCI-H2122 C) for MRTX849 and IN10018 for 72 hours. (Data represent Mean ± SEM, n = 3). Statistics analysis was done using unpaired student's T-test. D) Cell images from the 72 hour-drug combination test of MRTX849 and IN10018 on different cell lines. Scale bar = 100 µm. E) Cell images from the 72 hour-drug combination test of AMG510 and IN10018 on different cell lines. Scale bar = 100 µm. F) Images from cell clonogenic assay of 10 days-drug combination of MRTX849 and IN10018.

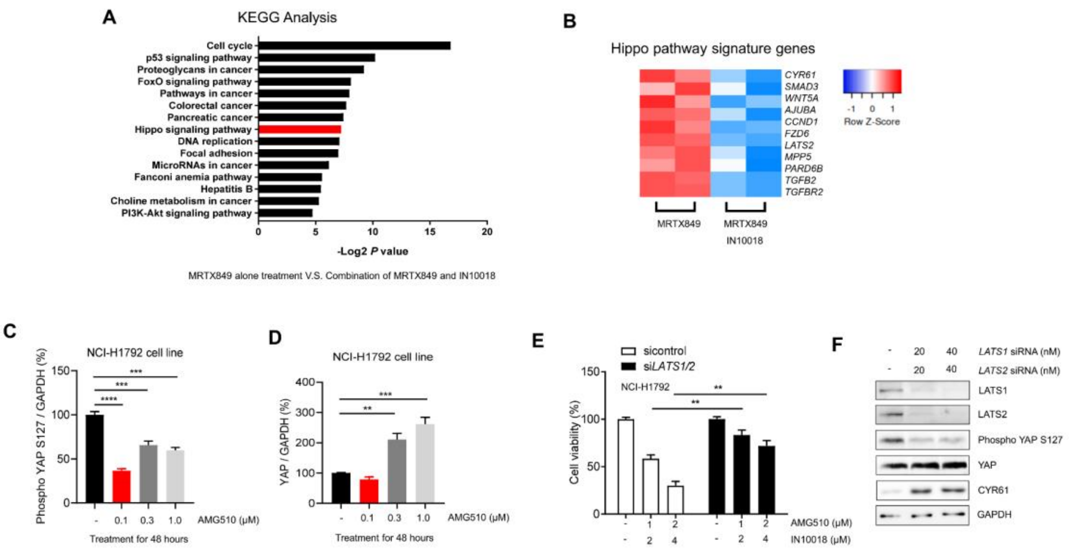

Figure S6. The transcriptome profiling analysis, YAP activity analysis, and cell viability rescue study of combination of AMG510 and IN10018 with *LATS1/LATS2* siRNAs. A) KEGG analysis of TOP 15 signal pathways which were downregulated by the combination of MRTX849 and IN10018 compared to MRTX849 monotherapy at 24 hours. B) Hippo pathway signature genes which were downregulated significantly by the combination of MRTX849 and IN10018 compared to MRTX849 monotherapy at 24 hours. C) The ratios of Phospho YAP S127 / GAPDH from Figure 5B. D) The ratios of YAP / GAPDH from Figure 5B. (Data represent Mean ± SEM, n = 4). Statistics analysis was done using one-way ANOVA. $**P < 0.01$, $***P < 0.001$, $****P < 0.0001$. E) The cell viability rescue study upon the combination treatment of AMG510 and IN10018 by knockdown of LATS1/LATS2 with siRNAs. The NCI-H1792 cells were transfected with control siRNA or *LATS1/LATS2* siRNAs. 24 hours later, the combination of AMG510 and IN10018 was added to the cell plates. The cell viability assay was conducted 72 hours post drug dosing. (Data represent Mean ± SEM, n = 4). Statistics analysis was done using unpaired student's T-test. $**P < 0.01$. F) Western blot data for testing the knockdown efficiency by *LATS1/LATS2* siRNAs at the study termination. Protein levels of Phospho YAP S127, total YAP, and CYR61 were checked to confirm the induction of YAP signaling by the transfection of *LATS1/LATS2* siRNAs on NCI-H1792 cell line.

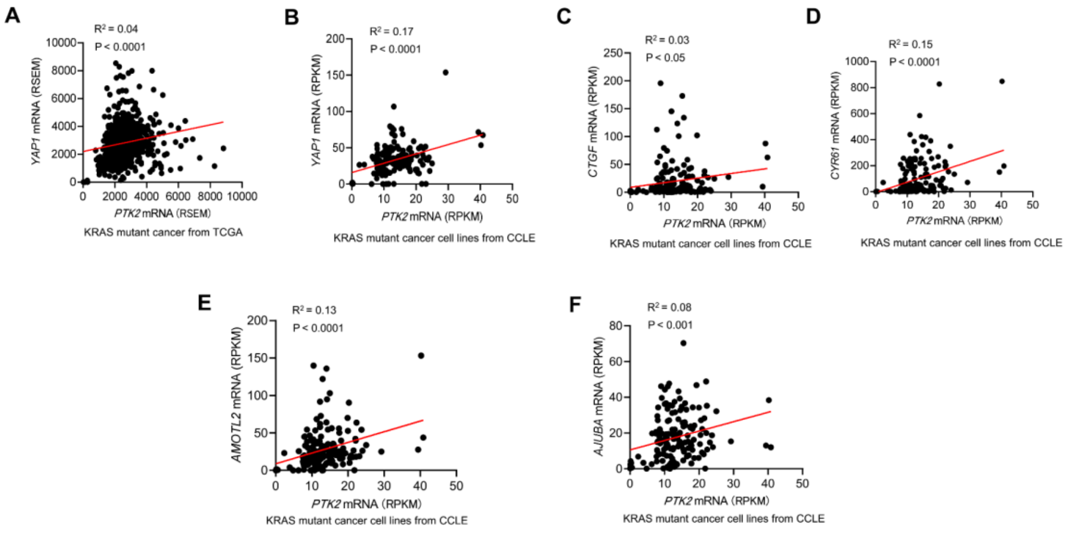

Figure S7. *PTK2* mRNA expression levels are positively correlated with *YAP1* mRNA and its downstream mRNA expression levels. A) Correlation analysis of *PTK2* and *YAP1* mRNA expression levels for KRAS mutant cancer patients from TCGA pan-cancer project (n = 734). Statistics analysis was done using slope coefficient test. R^2 and *P* values are shown on the graph. and CCLE analysis. B-F) Correlation analysis of *PTK2* and *YAP1* or its downstream mRNA (*CTGF*, *CYR61*, *AMOTL2*, and *AJUBA*) expression levels from KRAS mutant cell data of CCLE database (n = 174). Statistics analysis was done using slope coefficient test. R^2 and *P* values are shown on the graphs.

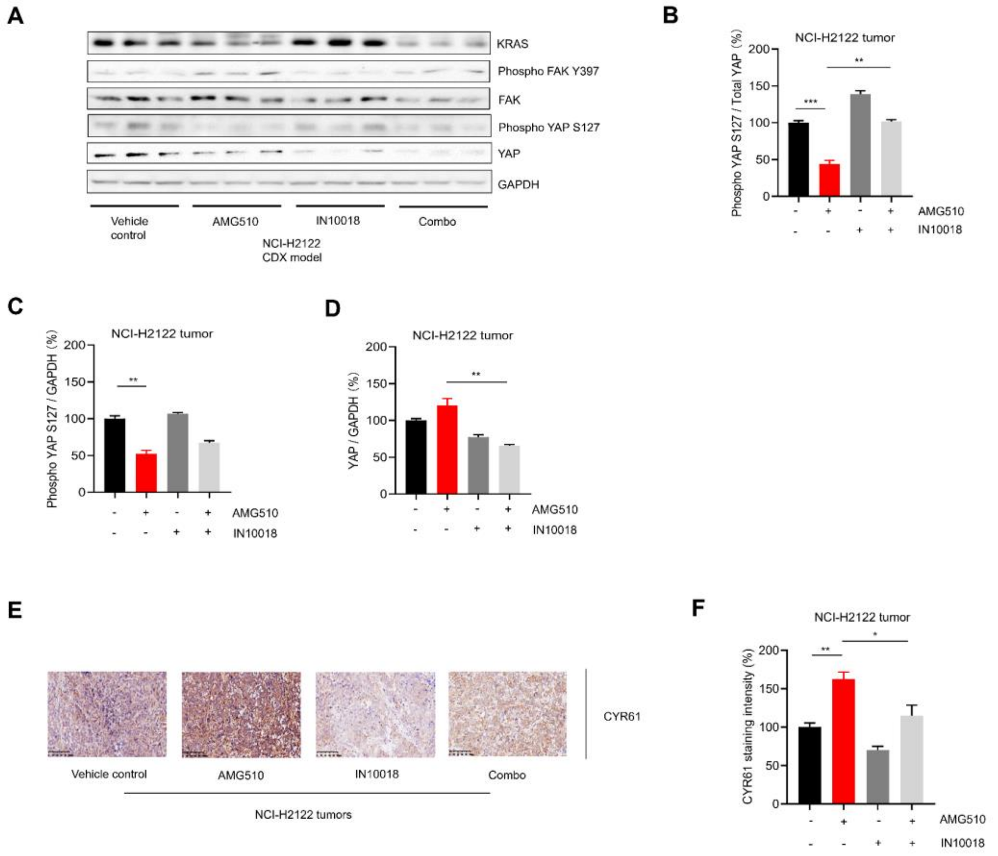

Figure S8. Western blot analysis and CYR61 expression levels of NCI-H2122 tumors from efficacy study. A) Western blot results. B-D) Ratios of Phospho YAP S127 / total YAP (B), Phospho YAP S127 / GAPDH (C), and Total YAP / GAPDH (D) for the western blot data of NCI-H2122 tumors (A). (Data represent Mean ± SEM, n = 3). Statistics analysis was done using unpaired student's T-test. ** $P < 0.01$, *** $P < 0.001$. E) CYR61 IHC staining for the NCI-H2122 tumors. Scale bar = 100 µm. F) Quantification of CYR61 expression levels of NCI-H2122 tumors from (E). (Data represent Mean ± SEM, n = 4). Statistics analysis was done using unpaired student's T-test. * $P < 0.05$, ** $P < 0.01$.

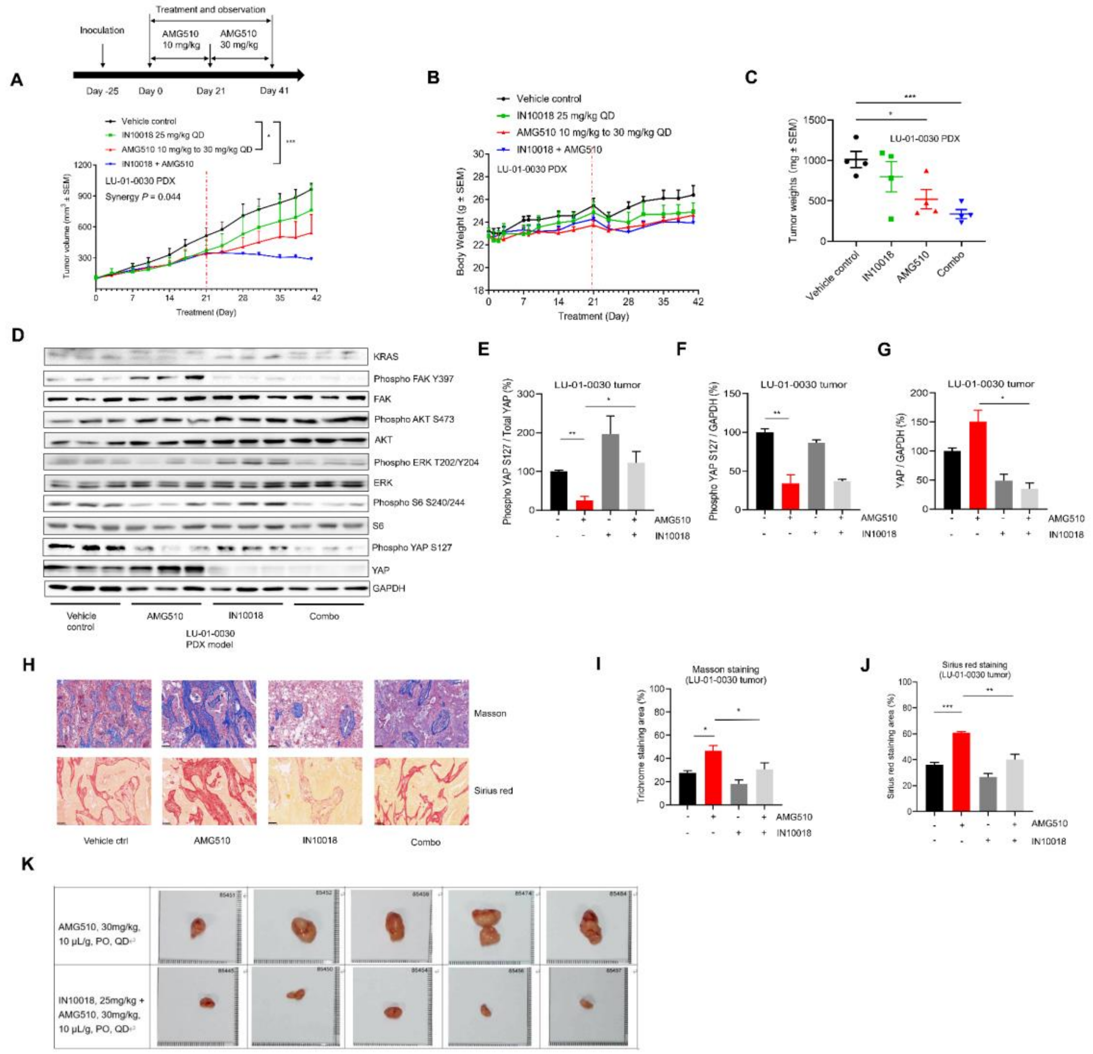

Figure S9. The combination of FAK and KRAS G12C inhibition exerts better tumor growth inhibition than every single treatment for NSCLC and CRC PDX models. A) Tumor growth curves of LU-01-0030 PDX model. (Data represent Mean ± SEM, n = 4, Synergy *P* value is shown). Statistics analysis was done using one-way ANOVA at the end of the study. **P* < 0.05, ****P* < 0.001. B) The body weight records of LU-01-0030 mice. C) Tumor weights at the end of the LU-01-0030 study. (Data represent Mean ± SEM, n = 4). Statistics analysis was done using one-way ANOVA. **P* < 0.05, ****P* < 0.001. D) Western blot results for the tumors from LU-01-0030 study. E-G) The ratios of Phospho YAP S127 / total YAP (E), Phospho YAP S127 / GAPDH (F), and total YAP / GAPDH (G) for different groups from LU-01-0030 study. (Data represent Mean ± SEM, n = 3). Statistics analysis was done using unpaired student's T-test. **P* < 0.05, ***P* < 0.01. H) Masson and Sirius red staining of the LU-01-0030 tumors. I) Measurements of Masson staining area of LU-010030 tumors. Scale bar = 50 µm. (Data represent Mean ± SEM, n = 3). Statistics analysis was done using unpaired student's T-test. **P* < 0.05. J) Measurements of Sirius red staining area of LU-01-0030 tumors. (Data represent Mean ± SEM, n = 3). Statistics analysis was done using unpaired student's T-test. ***P* < 0.01. ****P* < 0.001. K) Tumor images of CO-04-0070 PDX model treated with AMG510 and combination of AMG510 and IN10018.

Table S1. Primers and siRNA used in the study.

**Targets**	**Sequences**
CTGF	Forward: GTTTGGCCCAGACCCAACTA
	Reverse: GGCTCTGCTTCTCTAGCCTG
AJUBA	Forward: AGCCACCAGGTCCTTTCGTTCC
	Reverse: GGCATTGCTCTGCCCATAGATG
TGFB2	Forward: AAGAAGCGTGCTTTGGATGCGG
	Reverse: ATGCTCCAGCACAGAAGTTGGC
CYR61	Forward: CAGGACTGTGAAGATGCGGT
	Reverse: GCCTGTAGAAGGGAAACGCT
TEAD4	Forward: GAAGGTCTGCTCTTTCGGCAAG
	Reverse: GAGGTGCTTGAGCTTGTGGATG
AMOTL2	Forward: AGTGAGCGACAAACAGCAGACG
	Reverse: ATCTCTGCTCCCGTGTTTGGCA
TRIB3	Forward: GCTTTGTCTTCGCTGACCGTGA
	Reverse: CTGAGTATCTCAGGTCCCACGT
GAPDH	Forward: GTCTCCTCTGACTTCAACAGCG
	Reverse: ACCACCCTGTTGCTGTAGCCAA
Control siRNA	Sense: UUCUCCGAACGUGUCACGUTT
	Antisense: ACGUGACACGUUCGGAGAATT
YAP1 siRNA	Sense: GGUCAGAGAUACUUCUUAATT
	Antisense: UUAAGAAGUAUCUCUGACCTT
PTK2 siRNA	Sense: CCUGUAUGCCUAUCAGCUUTT
	Antisense: AAGCUGAUAGGCAUACAGGTT

LATS1 siRNA	Sense: GGUAGUUCGUCUAUAUUAUTT
	Antisense: AUAAUAUAGACGAACUACCTT
LATS2 siRNA	Sense: CUACCAGAAAGAGUCUAAUTT
	Antisense: AUUAGACUCUUUCUGGUAGTT

Table S2. Antibodies used in the study.

**Antibody**	**Vendor**	**Host species**	**Dilution factor**	**Catalog No.**
AKT	Cell signaling technology	Rabbit	1:1000	4691T
HRP-conjugated Alpha Tubulin	Proteintech	Mouse	1:5000	HRP-66031
CYR61	Proteintech	Rabbit	1:2000	26689-1-AP
ERK1/2	Cell signaling technology	Rabbit	1:2000	4695S
FAK	Cell signaling technology	Rabbit	1:1000	3285S
HRP-conjugated GAPDH	Proteintech	Mouse	1:5000	HRP-60004
HRP-conjugated Beta Actin	Proteintech	Mouse	1:5000	HRP-60008
KRAS	Sigma	Mouse	1:1000	SAB1404011
Lamin B1	Abcam	Rabbit	1:2000	ab133741
Histone H3	Abcam	Rabbit	1:2000	ab1791
S6	Cell signaling technology	Rabbit	1:2000	2217S

YAP	Cell signaling technology	Rabbit	1:1000	14074S
LATS1	Proteintech	Rabbit	1:500	17049-1-AP
LATS2	Cell signaling technology	Rabbit	1:1000	5888S
Phospho AKT S473	Cell signaling technology	Rabbit	1:1000	4060S
Phospho ERK1/2 T202/Y204	Cell signaling technology	Rabbit	1:2000	4370S
Phospho FAK Y397	Thermo fisher scientific	Rabbit	1:1000	44-624G
Phospho S6 S240/244	Cell signaling technology	Rabbit	1:2000	5364P
Phospho YAP S127	Cell signaling technology	Rabbit	1:1000	130083
Goat anti-rabbit IgG (H+L) secondary antibody HRP	Invitrogen	Goat	1:5000	31460
Goat anti-mouse IgG (H+L) secondary antibody HRP	Invitrogen	Goat	1:5000	31430
